# Cryo-EM Studies of Drp1 Reveal Cardiolipin Interactions that Activate the Helical Oligomer

**DOI:** 10.1038/s41598-017-11008-3

**Published:** 2017-09-06

**Authors:** Christopher A. Francy, Ryan W. Clinton, Chris Fröhlich, Colleen Murphy, Jason A. Mears

**Affiliations:** 1 Department of Pharmacology, Case Western Reserve University School of Medicine, Cleveland, OH, 44106 USA; 2 Center for Mitochondrial Diseases, Case Western Reserve University School of Medicine, Cleveland, OH, 44106 USA; 3 Cleveland Center for Membrane and Structural Biology, Case Western Reserve University School of Medicine, Cleveland, OH USA; 4 Crystallography, Max-Delbrück-Centrum for Molecular Medicine, Robert-Rössle-Straße 10, 13125 Berlin, Germany, Berlin, Germany; 5 Institute for Chemistry and Biochemistry, Freie Universität Berlin, Takustraße 6, 14195 Berlin, Germany, Berlin, Germany

## Abstract

Dynamins are mechano-chemical GTPases involved in the remodeling of cellular membranes. In this study, we have investigated the mechanism of dynamin-related protein 1 (Drp1), a key mediator of mitochondrial fission. To date, it is unclear how Drp1 assembles on the mitochondrial outer membrane in response to different lipid signals to induce membrane fission. Here, we present cryo-EM structures of Drp1 helices on nanotubes with distinct lipid compositions to mimic membrane interactions with the fission machinery. These Drp1 polymers assemble exclusively through stalk and G-domain dimerizations, which generates an expanded helical symmetry when compared to other dynamins. Interestingly, we found the characteristic gap between Drp1 and the lipid bilayer was lost when the mitochondrial specific lipid cardiolipin was present, as Drp1 directly interacted with the membrane. Moreover, this interaction leads to a change in the helical structure, which alters G-domain interactions to enhance GTPase activity. These results demonstrate how lipid cues at the mitochondrial outer membrane (MOM) can alter Drp1 structure to activate the fission machinery.

## Introduction

Mitochondria are essential membrane-bound compartments in eukaryotic cells that generate ATP and play a central role in activating apoptosis. Concurrently, mitochondria undergo continuous cycles of fission and fusion, creating a dynamic population of organelles capable of responding to physiologic signals^1, 2^. Mitochondrial division is essential to maintain cell health, but excessive fission has been observed in several diseases^3–7^. Despite this central role in mitochondrial health and disease, detailed insight into the fundamental mechanism of mitochondrial fission is lacking.

The key mediator of mitochondrial fission is dynamin-related protein 1 (Drp1), a large cytosolic GTPase (~80 kDa) belonging to the dynamin family of proteins. Drp1 localizes to mitochondrial fission sites and forms large oligomeric assemblies that are capable of inducing membrane constriction^8–11^. *In vitro*, Drp1 assembles on lipid templates, which can be used to mimic molecular interactions with the mitochondrial outer membrane (MOM), and these interactions facilitate self-assembly and stimulated GTPase activity^9, 12–14^. Based on previous studies with dynamin^15, 16^, this enhanced activity is believed to result from intermolecular G-domain dimerizations near the GTP-binding site, and a similar G-domain interaction was observed in Drp1 structural studies as well^17^. Additionally, extra sequence within the G-domain of Drp1 has been found to mediate a novel G-domain dimer^18, 19^. This interaction is stabilized through an 18 amino acid region called the 80 loop that is conserved in homologous mitochondrial fission dynamins^18^. Despite this previous work, the functional implications and the cellular contexts for these distinct G-domain dimers remains unknown.

**Figure 1:**
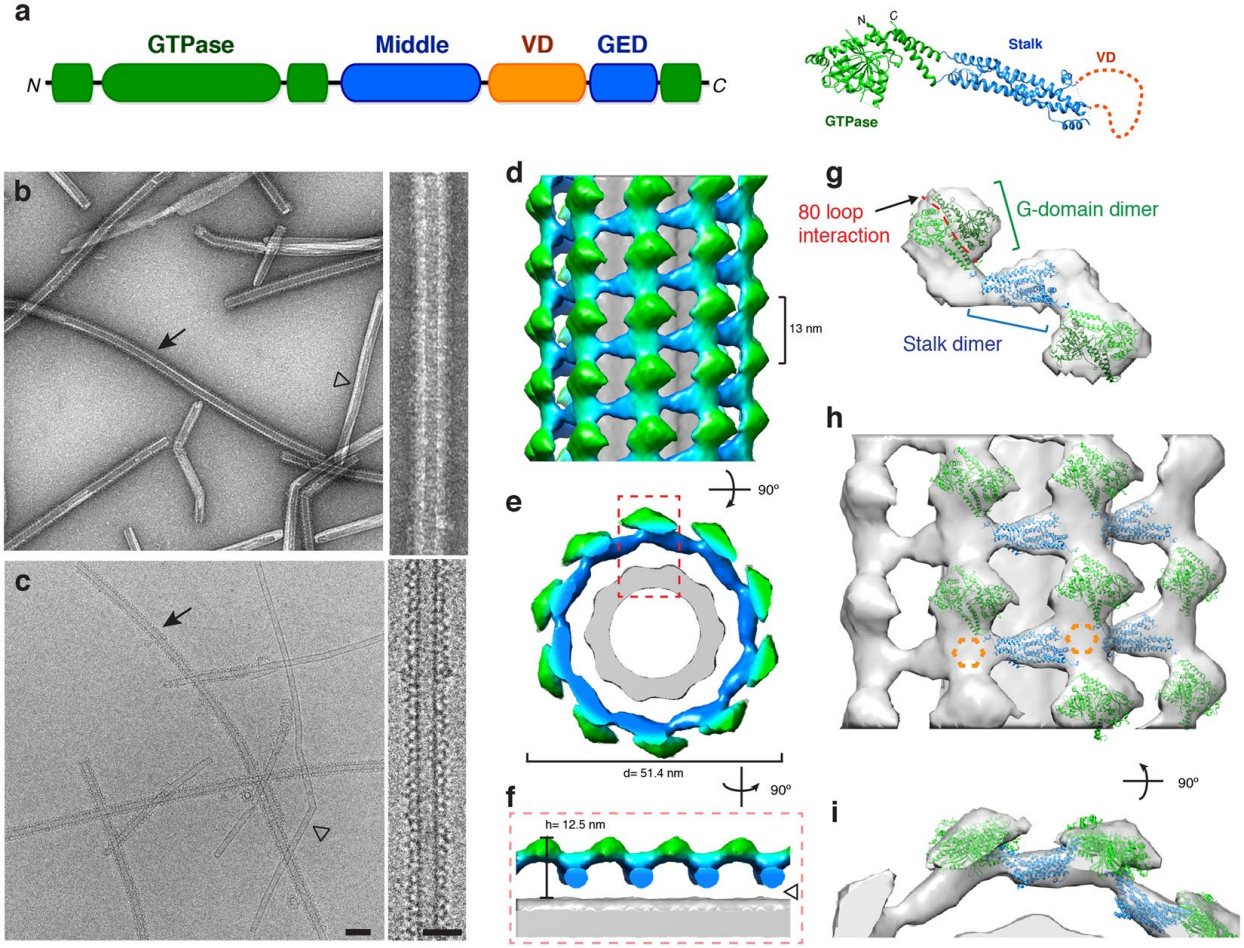
3D structure of Drp1 associated with a phosphatidylserine (PS) lipid template. (**a**) The primary sequence and tertiary structure (PDB ID: 4BEJ) of Drp1 highlights conserved domains: G domain (green), middle domain (blue), variable domain (orange) and GTPase effector domain (GED, blue). (**b**–**c**) Negative stain (**b**) and cryo-EM (**c**) images of Drp1 oligomerized in the presence of GMPPCP on galactosyl ceramide (GC) nanotubes containing phosphatidylserine (PS) at low (left; scale bar, 100 nm) and high magnifications (right; scale bar, 50 nm). Filled arrows indicate Drp1 decorated tubes, while open arrowheads indicate undecorated GC/PS tubes. (**d**–**e**) The 3D reconstruction of Drp1 on a GC/PS nanotube is presented. The helical pitch (13 nm) and diameter (51.4 nm) are indicated. (**f**) Cross-section of the 3D structure demonstrates the T-shaped architecture, and a gap between the protein and lipid is highlighted (open arrowhead). (**g**) The fitted structures of Drp1 GTPase (green) and stalk (blue) dimers are shown. The 80 loop interface (red) mediating G-domain dimerization is highlighted. (**h**) A side view of multiple Drp1 dimers fitted into the helical density. Unoccupied density is highlighted (dotted orange hexagon). (**i**) An end-on view of the same fitted structures.

In addition to G-domain interactions, Drp1 self-assembly is primarily driven via intermolecular stalk interactions^20^. The stalk is comprised of the middle domain and GTPase effector domain (GED, Fig. 1a), and several mutations in these regions have been shown to disrupt Drp1 oligomerization^20, 21^ and are associated with developmental defects in humans^22, 23^. Previously, crystallographic studies with dynamin, a related protein family member involved in endocytosis, revealed three important interfaces in the stalk. These interactions were also identified in cryo-EM structures of dynamin polymers on lipid templates^14, 16, 24, 25^. However, within the Drp1 crystal lattice, only one of these interfaces was conserved^20^. Consequently, it remains unclear how intermolecular Drp1 interactions drive assembly of the mitochondrial fission complex at membrane surfaces.

Between the middle and the GED sequences, an additional region called the variable domain (VD) bridges these domains similar to the pleckstrin homology (PH) domain in dynamin (Fig. 1a). This sequence has been shown to be an important regulatory component of Drp1 function^20^. Specifically, the VD serves as an autoinhibitory domain of Drp1 oligomerization as it maintains Drp1 in an assembly-limited cytosolic state^9, 26^. The VD is largely unstructured, and it has previously been shown to associate with negatively charged membranes, which relieves its inhibitory role to drive Drp1 self-assembly^9, 27^. In this way, the VD mirrors the lipid interaction properties of the dynamin PH domain, which binds to phosphoinositol-4,5-phosphate (PIP_2_) enriched membranes^28^. For Drp1, several recent studies support a significant VD interaction with the mitochondrial specific lipid, cardiolipin (CL), which promotes Drp1 assembly and augments GTPase activity^12, 13, 29, 30^. CL is most often found in the mitochondrial inner membrane (MIM), where it serves a critical role in electron transport chain function^31, 32^. Interestingly, CL translocation to the MOM has been reported at contact sites with the MIM^33^ and under stress conditions that induce mitophagy^34^. However, it is not known whether Drp1 stably associates with CL at the molecular level or how this interaction would convey enhanced activity to the GTPase domains of Drp1 polymers.

In order to address these critical questions about the mitochondrial fission complex, we have determined cryo-EM structures of human Drp1 helices on distinct membrane templates either containing the negatively charged phosphatidyl serine (PS) used in previous studies, or the mitochondrial specific CL. We found that distinct G-domain dimers were formed on each of these lipid templates, and that the magnitude of stimulated GTPase activity coincides with the mode of lipid-induced assembly of Drp1. In this way, changes in lipid composition can alter Drp1 structure and function. In addition to G-domain dimerization, we found that only one stalk interface was preserved within the lipid-induced Drp1 oligomers, and this produces a relatively extended polymer compared to dynamin. Lastly, we determined that the VD is required for CL interactions that induce functional assembly of Drp1 oligomers. In this way, CL microdomains at the surface of mitochondria could selectively trigger Drp1 activity to promote local constriction events. Collectively, these studies reveal the structural and functional consequences of Drp1 interactions with specific lipid membranes to advance our understanding of the mitochondrial fission complex.

## Results

### Dimerization through Intermolecular G-domain and Stalk Interactions Drive Drp1 Helical Oligomerization on PS Lipid Templates

Drp1 has been shown to assemble on negatively charged lipid membranes^9–12^, and PS has often been used as a model template given its ability to induce robust assembly of Drp1 and other dynamins. To build on these initial studies, we used negative stain electron microscopy (EM) to examine the structural properties of Drp1 polymers formed in the presence of PS liposomes. Although we observed robust formation of protein-lipid tubules, the diameters of these assemblies displayed a heterogeneous distribution (Supplementary Fig. 1b, 119 ± 49 nm), which highlights the inherent ability of Drp1 to accommodate diverse membrane geometries. As shown previously^9, 20^, the addition of a non-hydrolyzable GTP analogue, GMPPCP, led to a moderate decrease in the average diameter (108 ± 44 nm), and we refer to this conformation of Drp1 as a stabilized phase (Supplementary Fig. 1a), since lipid and nucleotide interactions limit the formation of larger helical polymers. Still, this pre-constriction state presents a broad distribution of diameters (Supplementary Fig. 1c). Therefore, galactosyl ceramide (GC) was used to generate lipid nanotubes with a relatively fixed geometry and isolate Drp1 polymers in a pre-constricted state. Incorporation of 30 mol% PS into the GC nanotubes (GC/PS) generated a uniform lipid template with sufficient negative charge to recruit Drp1. These templates enriched a population of Drp1-lipid tubules with smaller diameters that represented a subset of the distribution observed using PS liposomes (Supplementary Fig. 1d, 59 ± 4 nm). This enhanced homogeneity of Drp1 oligomers formed on GC/PS templates in the presence of GMPPCP provided an optimal sample for cryo-EM studies to examine protein-lipid interactions.

Drp1 oligomers on GC/PS templates were readily observed using negative stain and cryo-EM (Fig. 1b–c, respectively). The laddering pattern displayed on the surface of the lipid nanotubes demonstrates Drp1 helical decoration on this membrane surface (closed arrow). Undecorated nanotubes were less abundant (Fig. 1b–c, open arrowhead), and these were disregarded for structural studies of the protein-lipid interaction. The 3D structure of Drp1 on a PS-containing membrane was determined using an Iterative Helical Real Space Reconstruction algorithm (IHRSR)^35, 36^ (Fig. 1d). The helical lattice had a pitch of 13 nm and a right-handed symmetry (Fig. 1d, Supplementary Fig. 2b,c, more information available in Online Methods). Dimeric Drp1 forms the fundamental asymmetric unit^12^, and 10 dimers comprised one turn of the helix, which represents a novel Drp1 helical conformation.

From the final reconstruction, the globular GTPase domains (green) are clearly discernable from the stalk regions (blue) of Drp1, and the protein assembly forms a characteristic T-shaped architecture similar to other dynamins (Fig. 1f)^14, 24, 25^. Interestingly, there appears to be no stable Drp1 contact with the lipid surface (Fig. 1e,f, open arrow head), which is consistent with previous studies using the yeast homolog of Drp1^14^. This “gap” may be due to helical averaging of flexible protein segments that weakly interact with the lipid surface. Still, the interaction between Drp1 and the PS template is not stable.

Upon docking the Drp1 crystal structures into the density, it was found that G-domain and stalk interactions were driving the helical assembly (Fig. 1g,h). Interestingly, the canonical G-G interaction, proximal to GTP-binding sites, that has been demonstrated with dynamin did not fit well into the EM density (Supplementary Fig. 3). Rather, the G-domain dimer formed via the 80 loop^18^ closely matched the density when fitted (Fig. 1h,i). This unique interaction is still consistent with the stalk occupying the middle radial density (blue) that dives towards the lipid membrane. Within this region, additional protein density was apparent (Fig. 1h, dotted orange hexagon), and we attribute this to VD interactions near the stalk, comparable to interactions between the PH domain and stalk of dynamin^37^. In this conformation, interactions between the VD and lipid would likely be transient, which is consistent with the weak membrane association.

### Cardiolipin Nanotubes Drive Functional Assembly of Drp1

**Figure 2:**
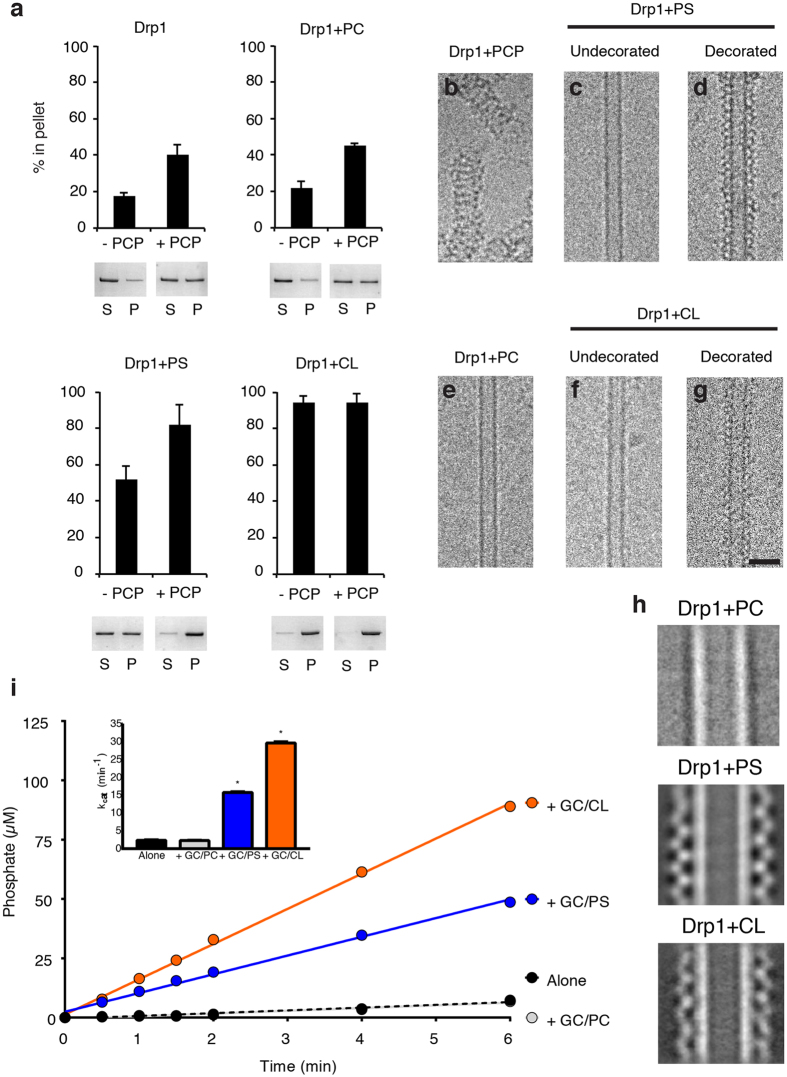
Drp1 recruitment and activation is enhanced with cardiolipin (CL) nanotubes. (**a**) Sedimentation analysis are presented for Drp1 alone, incubated with phosphatidylcholine nanotubes (GC/PC), phosphatidylserine nanotubes (GC/PS) and cardiolipin nanotubes (GC/CL) in the absence and presence of GMPPCP (−PCP and +PCP, respectively, n = 3/sample. Representative supernatant (S) and pellet (P) fractions are shown. Cryo-EM images are shown of Drp1 in the presence of GMPPCP (**b**), PS nanotubes (undecorated, (**c**), and decorated, (**d**)), PC nanotubes (no protein decoration observed, (**e**)), and CL nanotubes (undecorated, (**f**), and decorated, (**g**)). Scale bar, 50 nm. (**h**) 2-D class averages of Drp1 + GC/PC, Drp1 + GC/PS and Drp1 + GC/CL are presented. (**i**) A GTP hydrolysis assay displays the amount of phosphate released over time for Drp1 alone (black) and incubated with different nanotubes (GC/PC, gray; GC/PS, blue; GC/CL, orange, n = 3/sample). Measured GTPase activities (kcat) are shown (inset).

There have been several studies demonstrating the ability of CL to robustly stimulate Drp1 GTPase activity^12, 29^ through direct interactions with Drp1^30^. To further explore the recruitment and enzymatic activity using structural lipid templates, we utilized sedimentation assays, cryo-EM and GTPase assays to measure Drp1 interactions on several nanotubes with distinct lipid compositions. Alone in solution, Drp1 does not sediment with medium-speed centrifugation (17% in pellet, Fig. 2a), which is consistent with the smaller oligomers sampled in solution^12^. Conversely, Drp1 sedimentation increases after the addition of GMPPCP (41% in pellet) (Fig. 2a), which is consistent with the formation of larger protein spirals in solution (Fig. 2b). The addition of neutral charged lipid nanotubes containing 12 mol% phosphatidylcholine (PC) did not increase Drp1 sedimentation when compared with the protein alone (22% and 45% in the pellet in the absence and presence of GMPPCP, respectively) (Fig. 2a). Cryo-EM images revealed that Drp1 could not assemble on the GC/PC lipid nanotubes with or without GMPPCP (Fig. 2a,e). Therefore, the curvature of the lipid nanotubes alone was not sufficient for Drp1 recruitment and polymerization. Conversely, the addition of GC/PS and GC/CL (12 mol% CL) initiated robust assembly of Drp1 oligomers as larger sedimentation values were observed (52% and 92% in the pellet, respectively). Addition of GMPPCP enhanced oligomerization with the PS nanotubes (an increase to 82% in the pellet), and we attribute this observation to enhanced lipid oligomerization as we found increased decoration on the nanotubes and few nucleotide-induced spirals in the background using cryo-EM. Conversely, the CL nanotube appeared to be saturated whether nucleotide was present or not (94% and 92% in the pellet, respectively), which demonstrates a stronger interaction for Drp1 with CL. Images of the Drp1-lipid tubule segments exhibited an ordered protein assembly around the lipid core (Fig. 2d,g), and this pattern was noticeably absent for undecorated nanotubes (Fig. 2c,e,f). Two dimensional class averages further illustrate these differences, as Drp1 uniformly decorated nanotubes containing PS and CL, while PC nanotubes were undecorated (Fig. 2h). The protein lattice on the CL nanotubes also appears to adopt a distinct conformation when compared with the PS template.

To ensure that the lipid nanotubes were forming functional Drp1 complexes, we utilized a phosphate release GTPase assay^38^. Drp1 in solution displayed a basal activity (2.3 min^−1^), and the addition of GC/PC lipid did not alter this measurement (2.3 min^−1^), which is consistent with the lack of lipid-induced protein self-assembly (Fig. 2i). Consistent with previous studies, GC/CL stimulated Drp1 activity (30 min^−1^, a 13-fold enhancement) more than did GC/PS (16 min^−1^, a 7-fold increase) (Fig. 2i). Nevertheless, the negatively charged nanotube templates were able to induce functional Drp1 oligomerization. In addition, the increased stimulation in GTPase activity and the differences in 2D class averages suggested that CL formed a distinct Drp1 helical structure that could represent an alternative functional state dependent on the lipid composition of the membrane.

### Cardiolipin Interactions Stabilize an Active Helical Drp1 Conformation

**Figure 3:**
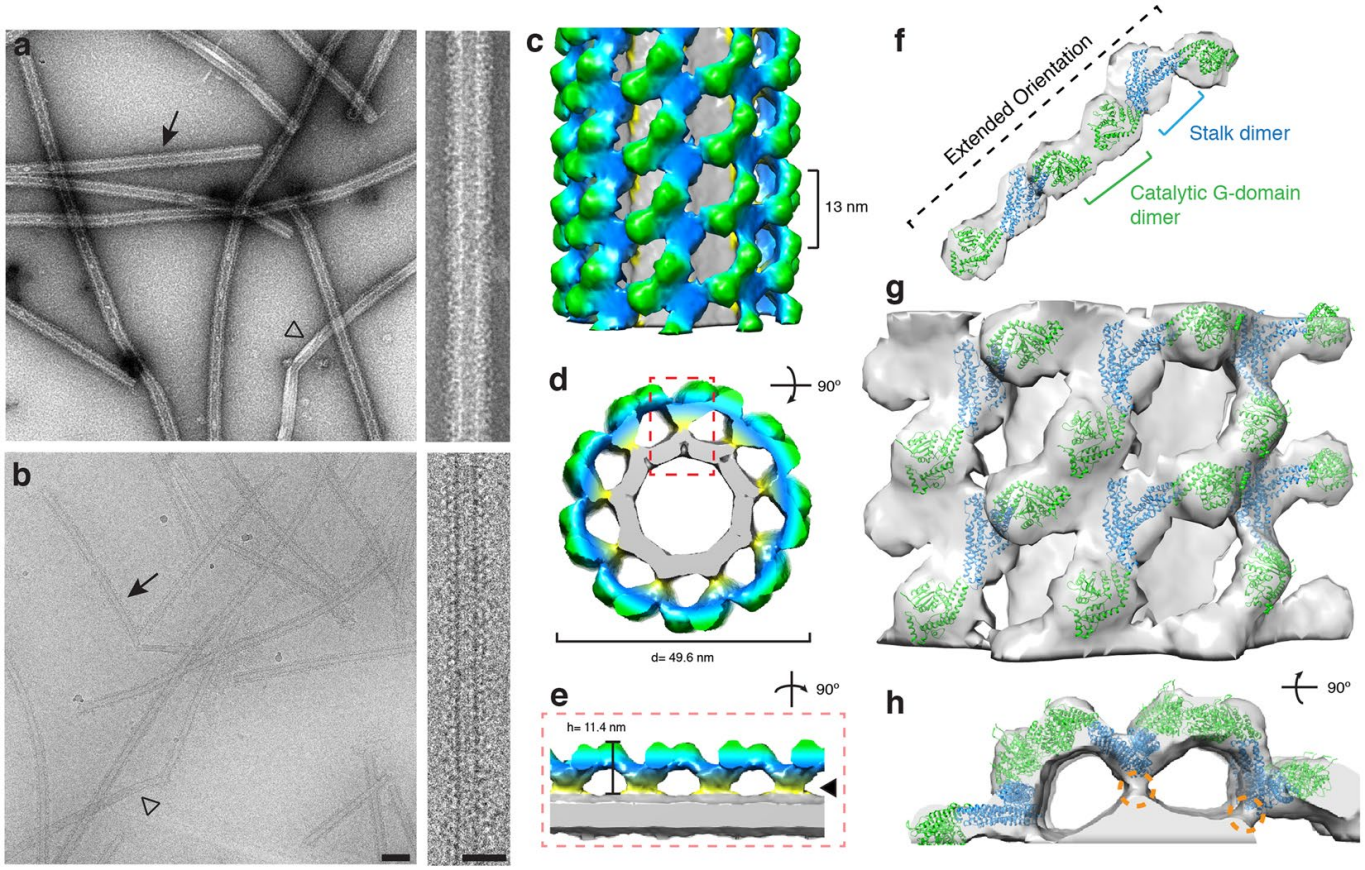
3D structure of Drp1 bound to a cardiolipin (CL) lipid template. (**a**–**b**) Negative stain (**a**) and cryo-EM (**b**) images of Drp1 oligomerized in the presence of GMPPCP on galactosyl ceramide (GC) nanotubes containing cardiolipin (CL) at low (left; scale bar, 100 nm) and high magnifications (right; scale bar, 50 nm). Filled arrows indicate Drp1 decorated tubes, while open arrowheads indicate undecorated GC/PS tubes. (**c**–**d**) The 3D reconstruction of Drp1 on a GC/CL nanotube is presented. The helical pitch (13 nm) and diameter (49.6 nm) are indicated. (**e**) A cross-section of the 3D structure is presented and the stabilized Drp1-lipid interaction is highlighted (filled arrowhead). (**f**) The fitted structures of Drp1 GTPase (green) and stalk (blue) dimers are shown. The G domain dimers interact through a different catalytic interface when compared to the GC/PS structure. (**g**) A side view of multiple Drp1 dimers fitted into the helical density is presented. (**h**) An end-on view of the same fitted structures. Density contacting the lipid surface (orange dotted circle) likely represents VD interactions.

Since CL recruits and activates Drp1 more robustly than other negatively charged liposomes, we explored the structure of Drp1 helices on CL nanotubes to identify potential differences. Negative stain and cryo-EM images revealed that the Drp1-decorated CL nanotubes were largely comparable to the Drp1 + GC/PS sample (Fig. 3a,b vs. Fig. 1b,c). However, closer examination of the cryo images revealed that the Drp1-CL oligomer appeared to associate more strongly with the lipid surface (Figs 2h, 3b, right panel). Using the same IHRSR method described previously, we determined the 3D structure of Drp1 polymers on CL nanotubes in the presence of GMPPCP (Fig. 3c). The oligomer was also found to have a right-handed symmetry (Supplementary Fig. 2) and a similar helical pitch of 13 nm. While the Drp1 dimer still formed the fundamental, repeating unit within the helical lattice, only 9 subunits were found to comprise one turn of the helical lattice (vs. 10 subunits for the Drp1-PS helices). This change coincides with a different Drp1 helical conformation, and the G-domain (green) and stalk (blue) regions of the dimers assumed distinct orientations. Most strikingly, this altered assembly was promoted through changes at the lipid surface. Specifically, the Drp1 polymer forms a stable, direct interaction with the CL nanotube. In fact, no gap was found between the protein and membrane, and a strong protein density was observed proximal to the lipid tubule (Fig. 3d,e, filled arrow head).

**Figure 4:**
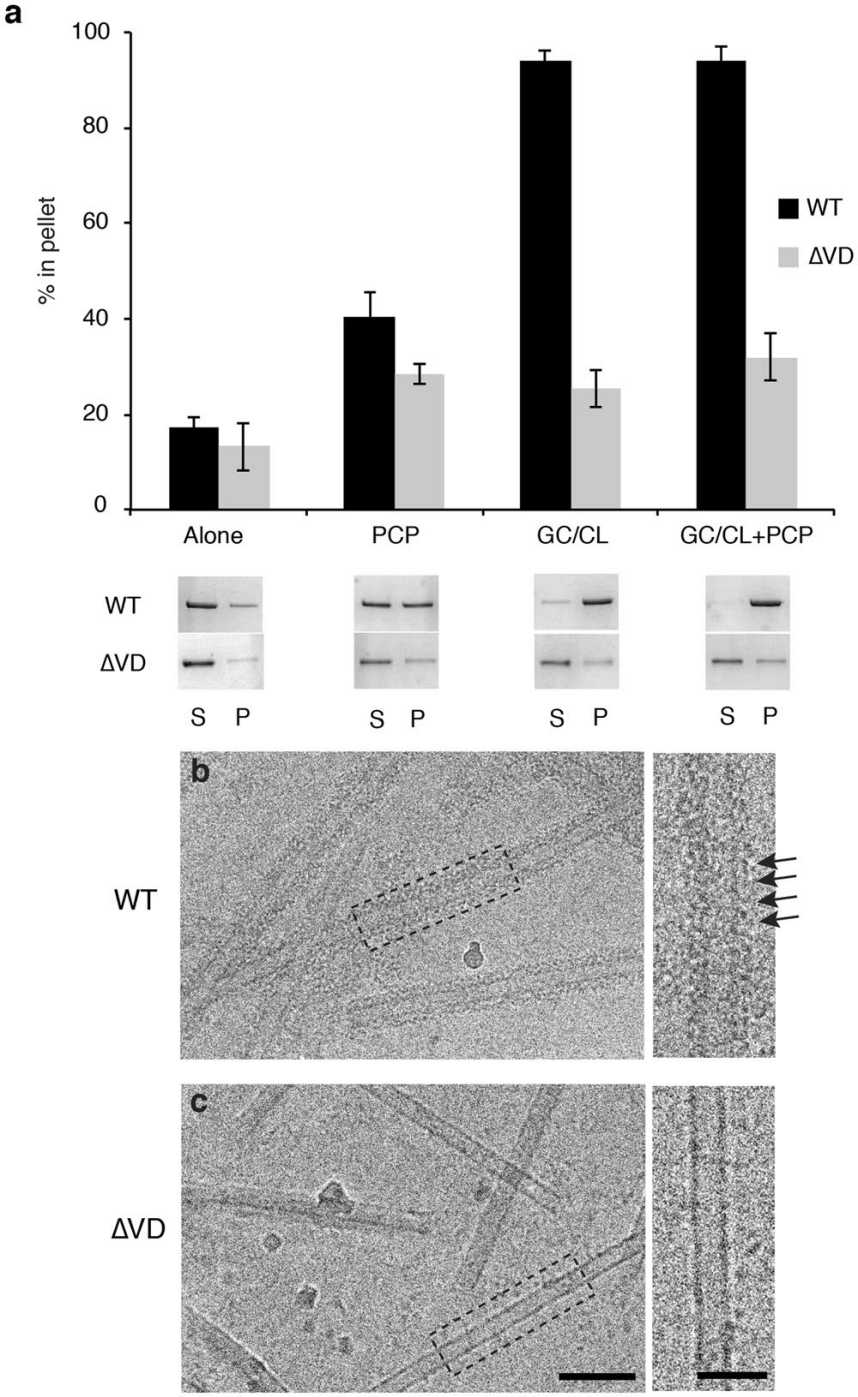
Removal of the variable domain (VD) abolishes Drp1 oligomerization with CL nanotubes. (**a**) Sedimentation was measured to assess the oligomeric state of wild-type (WT, black) and ΔVD (grey) Drp1 alone, in the presence of GMPPCP, in the presence of CL nanotubes (GC/CL) and in the presence of CL nanotubes with GMPPCP (GC/CL + PCP). Representative supernatant (S) and pellet (P) gel samples are shown. (**b**) A representative cryo-EM image demonstrates WT Drp1 decoration of the GC/CL nanotube in the presence of GMPPCP, the arrowheads indicate rungs of the protein helix. (**c**) Under the same conditions ΔVD did not decorate the nanotubes. Scale bar, 100 nm. Insets display the boxed region on the micrograph. Scale bar, 50 nm.

The density contacting the lipid surface was attributed to the VD, which was deleted in previous crystallographic studies (Fig. 3h, orange dotted circle). To confirm the role of the VD in direct association with the CL nanotube, we utilized a ∆VD Drp1 mutant lacking this region^20^. Sedimentation assays were performed with the ∆VD mutant to examine lipid association and protein oligomerization. Compared to WT, ∆VD sedimentation was greatly reduced on the GC/CL template (25% in the pellet, Fig. 4a) and this value only slightly increased when GMPPCP was added (32% in the pellet, Fig. 4a). This is consistent with previous studies that have shown diminished CL interactions when the VD was mutated^30^. Using cryo-EM, we also confirmed that ∆VD did not decorate CL nanotubes (Fig. 4b,c **)**. Collectively, these data demonstrate that the VD is essential for Drp1 association with CL lipids leading to helical polymerization.

Docking of the Drp1 crystal structure revealed that connection between the stalk dimers is maintained through a similar interaction, but each stalk was tilted toward the membrane surface, likely due to interactions with the lipid (Fig. 3f–h). Interestingly, the G-domain interactions within the Drp1-CL oligomers were found to undergo a large conformational rearrangement when compared to the GC/PS model, which results in a more extended dimer orientation. This orientation would support G-G interactions adjacent to the GTP-binding sites, which is consistent with structures observed in previous dynamin studies^15^ (Fig. 3f,g). This interaction also correlated with enhanced GTPase activity, which implies that direct interaction with a CL membrane can rearrange the Drp1 polymer to promote GTP hydrolysis and subsequent constriction.

**Figure 5:**
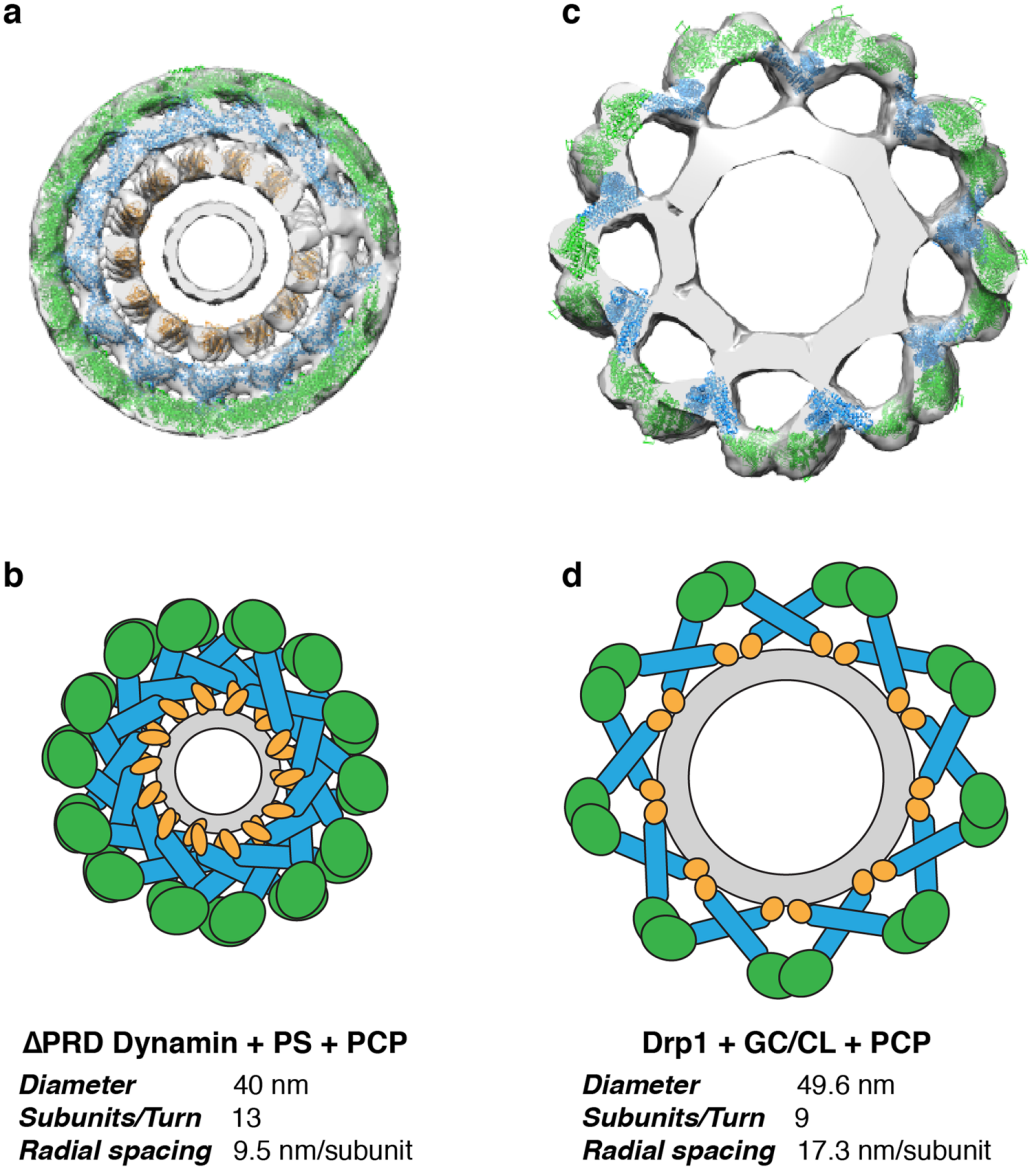
Comparison of the lipid-bound Dynamin and Drp1 polymers. (**a**) The 3D cryo-EM reconstruction of the ΔPRD dynamin helical oligomer formed in the presence of PS liposomes and GMPPCP is presented with the GTPase (green), stalk (blue) and PH (yellow) domains fitted16. (**b**) The tight helical packing of dynamin is illustrated and the helical parameters are shown. (**c**) The 3D cryo-EM reconstruction of the Drp1 helical oligomer formed in the presence of CL-containing nanotubes and GMPPCP is presented with the GTPase (green) and stalk (blue) domains (PDB ID: 4BEJ) fitted. (**d**) The more expanded helical packing of the Drp1 helix is illustrated and the helical parameters are shown.

Comparing our cryo-EM reconstructions to other dynamin family members, one striking difference is the greater spacing within the helical lattice (Fig. 5 **)**. In fact, the lipid nanotube is clearly visible beneath the protein coat. In the dynamin helical structure, the protein layer is much more densely packed^24, 25, 39^ (Fig. 5a,b). The lack of additional stalk interfaces within the Drp1 oligomer leads to an expanded assembly (Fig. 5c,d). To this point, Drp1 dimers traverse a greater radial path length, as there are fewer subunits per turn (GC/PS: ~10 subunits/turn, GC/CL: ~9 subunits/turn) when compared with ∆PRD-dynamin in a similar state (~13 subunits/turn). This topology may be necessary for Drp1 to remain dynamic and accommodate various lipid curvatures. Nonetheless, stalk and G-domain dimerization appear to be the minimal interfaces required for active Drp1 polymerization.

## Discussion

In this study, we have identified key assembly factors that drive Drp1 helical oligomerization on lipid templates in the presence of nucleotide. As shown previously^9, 20^, Drp1 is capable of forming protein lipid tubules with a range of diameters (Supplementary Fig. 1). To limit heterogeneity for structural studies, we decorated lipid nanotubes with Drp1 in the presence of GMPPCP to maintain more uniform helical diameters. These represent a subset of narrow oligomers that Drp1 formed when remodeling anionic liposomes (Supplementary Fig. 1). Drp1 interaction with negatively charged surfaces agrees with *in vivo* data, where Drp1 associates with several anionic membranes and structures within the cell, including mitochondria, peroxisomes, microtubules and actin^20, 40–42^. Therefore, these structural studies reveal how Drp1 interactions with negatively charged membranes influence its structure and function.

Importantly, these structures represent a pre-constricted GTP-bound state (Supplementary Fig. 1a). When GTP hydrolysis is occurs, Drp1 constriction leads to a transient association with the membrane, and therefore, this state is difficult to isolate for structural studies. Moreover, GC nanotubes are rigid templates that would limit radial constriction following hydrolysis. Therefore, future studies examining the constricted phases would require the use of a malleable lipid template. In these studies, the GC template provides a platform to isolate Drp1 interactions with negatively charged lipids at the membrane surface.

Regardless of whether PS or CL was mixed with GC to form Drp1-lipid structures, the fundamental building block within the Drp1 polymer is a dimer. This is consistent with previous studies demonstrating preferential assembly of dimers into larger oligomeric fission complexes^12^. Moreover, within the GC/PS and GC/CL models, we can see that the stalk interface that drives dimerization in all dynamins is conserved, and mutagenesis in this region completely ablates Drp1 oligomerization^20^. Interestingly, oligomerization of other dynamin family members are primarily driven through additional stalk interactions^14, 25, 39, 43–45^. However, these other interfaces were not observed in our structures, owing to the greater spacing within the helical lattice (Fig. 5a–d). We cannot exclude important roles for additional Drp1 interfaces. Proposed interaction sites near the bottom of the stalk (previously termed interface 3) and between Drp1 dimers (interface 4) may be important for solution multimers or for distinct helical oligomers. It appears that Drp1 can assume different modes of assembly, which may be enforced by specific interactions at the surface of mitochondria.

**Figure 6:**
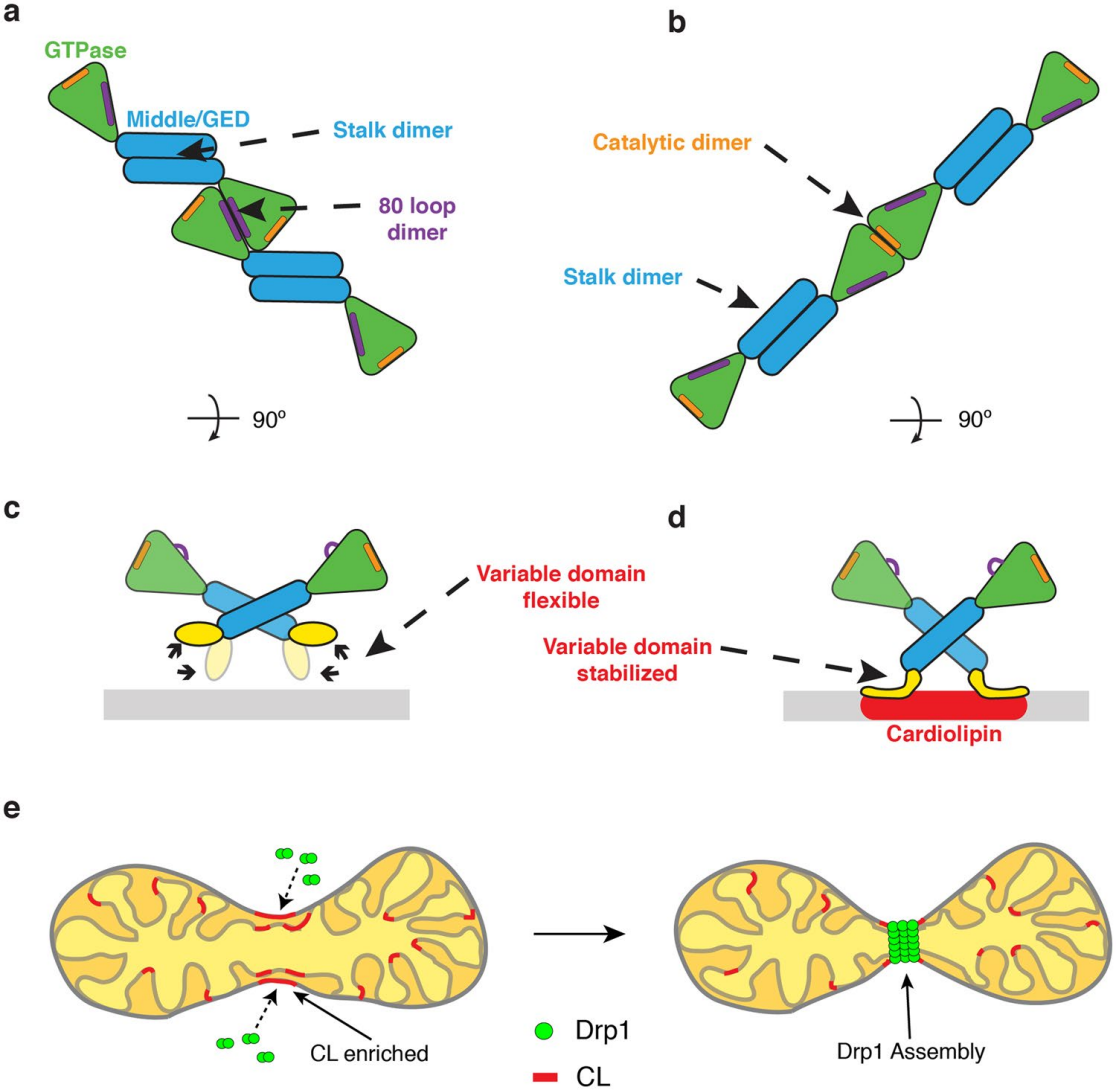
Cardiolipin interactions trigger active Drp1 assembly. (**a**) A model illustrates the Drp1 architecture on a GC/PS template. Stalk and 80 loop (purple) G-domain dimerization drive helical assembly. (**b**) A separate model illustrates the Drp1 architecture on a GC/CL template. Stalk and distinct G-domain dimerizations near the GTP binding pocket (orange) promote assembly of a more active polymer in the presence of CL. (**c**) On a GC/PS template, the variable domain of Drp1 interacts weakly with the membrane surface. (**d**) On a GC/CL template, the variable domain is stabilized on the membrane surface, which induces a pivot at the stalk interface to transmit a specific lipid signal at the membrane to the peripheral G domains where an activate conformation is formed. (**e**) A model depicts Drp1 interactions with CL at the surface of mitochondria where this unique lipid activates Drp1 function at defined sites primed for mitochondrial fission.

Intermolecular G-domain contacts are formed in the presence of nucleotide to further build the helical polymer (Fig. 5c,d). Despite the importance of the stalk domains in oligomerization, we found that the G-domain interactions play a significant role in driving Drp1 helical assembly as well. Intriguingly, the G-domain dimers formed in the GC/PS structure were distinct from those seen in the GC/CL structure. More specifically, we found that the 80 loop G-domain dimer, solved in previous crystal studies^18^, best fits in the G-domain density on the PS membrane (Fig. 6a). Conversely, the G-domains are held in a “front-to-front” orientation in the GC/CL model, and this leads to an enhanced GTPase activity through a mechanism similar to G-domain dimerization seen in other dynamin structures^46^ (Fig. 6b). Specifically, these G-domain dimers are juxtaposed to allow for intermolecular interactions near the nucleotide-binding site. This conformational change in the presence of CL suggests that the VD can sense lipid microdomains and transduce a conformational change from the base of the molecule through the stalk to reorient G-domain interactions at the periphery of the contractile apparatus (Fig. 6d). To this point, cardiolipin has also been shown to preferentially interact with flexible protein domains^47^, and the VD is largely disordered. Drp1 interactions with CL in the cell would likely occur at specific sites, as CL is known to be enriched at contact sites between the MOM and MIM^33^ and externalized CL interacts with several cytosolic proteins on the MOM including Bax, tBid and LC3 proteins involved in mitophagy and apoptosis^34^. Under stress conditions, CL is significantly enriched on the MOM prior to apoptosis^48, 49^. Thus, Drp1 assemblies can respond to specific molecular cues, such as CL, to alter functional processivity and induce mitochondrial fission (Fig. 6e).

The Drp1 helical structure on GC/PS membranes is in a different conformation with limited activity compared to GC/CL. Strikingly, this complex does not strongly contact the lipid membrane, similar to what was observed with the yeast mitochondrial fission protein Dnm1^14^. Moreover the G-domains in the GC/PS structure dimerize through a distinct interface near the 80 loop. This stretch of 16 amino acids in the 80 loop is unique to Drp1 and other dynamin-related proteins involved in mitochondrial fission. We propose that this novel interaction may represent an additional regulatory interface. Specifically, Drp1 would assemble in a primed conformation until additional signals induce a conformational change in the VD that propagates through the stalk and G-domain to promote targeted Drp1 constriction. In cells, Drp1 has been shown to sample the MOM in oligomeric forms without inducing a fission event^13, 41^. Therefore, the GC/PS polymer may represent a sampling assembly, and additional cues, including CL interactions, alter Drp1 conformation to promote GTP hydrolysis leading to Drp1 constriction and subsequent fission. Therefore, the VD may be important for fission by correctly targeting Drp1 to CL enriched areas on the mitochondria, and stabilizing Drp1 in a conformation that promotes mitochondrial constriction and subsequent fission.

Mitochondrial fission partner proteins, including Mff, MiD49, MiD51 and Fis1, may also act as physical cues that promote functional assembly of mitochondrial fission complexes, similar to CL. The structure of the Drp1 helix has sufficient spacing to accommodate partner proteins within the polymer lattice, which could further explain the unique helical symmetry. Previous studies have shown interactions with Mff stimulate Drp1 GTPase activity^27, 29^, while interactions with MiD51 have been shown to reduce Drp1 GTPase activity^50^. Therefore, it is likely that this additional regulation with partner proteins coincides with lipid cues on the membrane surface to modulate Drp1 recruitment and function.

In the cell there are many physiologic cues that recruit specialized proteins so they can perform their distinct function. One example includes PS, which is externalized to the outer leaflet of the plasma membrane during apoptosis and acts as an ‘eat-me’ signal. PS-specific receptor proteins bind externalized PS and induce eventual apoptosis^51^. In a similar fashion, CL may act as a ‘divide-me’ signal on the MOM, providing the cue for activation of the Drp1 polymer. In disease, CL externalization may be enhanced resulting in accelerated scission and enhanced apoptosis through a more efficient mitochondrial division process. In the future, this Drp1-CL interaction could be an attractive therapeutic target.

Previously, nucleotide induced conformational changes of dynamin have been demonstrated, but in this study we show that specific lipid interactions can specifically induce conformational changes that enhance Drp1 function. Interestingly, dynamin may also undergo lipid specific conformational changes, as previous work has shown greater stimulation on PIP2 containing membranes compared to PS membranes^52^. Therefore, the ability of dynamins to respond to lipid signals may be conserved. Overall, this work advances our understanding of the potential cues and molecular mechanisms necessary to induce Drp1 mediated mitochondrial fission.

## Methods and Materials

### Drp1-Lipids Preparations

Human Drp1 (UniProtID: O00429–3, residues 1–710, isoform 2) was expressed and purified as described previously^20^ (more detail in Supplementary Methods). All lipids were purchased from Avanti Polar Lipids Inc. (Alabaster, AL). The liposomes used in this study included 1,2-dioleoyl-sn-Glycero-3-[Phospho-L-Serine] (DOPS), heart bovine cardiolipin (CL), 1,2-dioleoyl-sn-glycero-3-phosphocholine (DOPC) and D-Galactosyl-β1-1′-N-Nervonoyl-D-erythro-Sphingosine (C24:1 β-D-Galactosyl Ceramide) (GalCer). Specific lipids in this study were dissolved in chloroform and dried under a nitrogen stream. For structural studies, 30 mol% DOPS was mixed with GalCer (GC/PS), and separately 12 mol% CL was mixed with GalCer (GC/CL). Additionally, 12 mol% DOPC in GalCer (GC/PC), GC/PS and GC/CL were used for sedimentation and GTPase assays. The dried lipid was stored in vacuum overnight and rehydrated in 37 °C PBS buffer, followed by occasional vortexing. Assembly buffer was used to resuspend lipids for GTPase assays.

Drp1 was resuspended to 0.4 mg/ml in PBS buffer (10 mM phosphate buffered saline, 138 mM NaCl, 2.7 mM KCl, 10 mM BME, pH 7.4). Lipid nanotubes with a specific composition were added to the mixture to a final concentration of 0.4 mg/ml (450 uM). The suspension was incubated at RT for 5 minutes, then 1 mM GMPPCP (Sigma) was added. The GC/PS and GC/CL samples in the presence of GMPPCP were incubated for 30 minutes to several hours before making EM grids.

### Electron Microscopy Studies

For negative stain, samples were stained with 2% (w/v) uranyl acetate (Polysciences, Inc.) on carbon-coated grids. For cryo-EM preparations, samples were applied to holey carbon grids (R3.5/1, Quantifoil). After a 1 minute incubation, the grid was blotted with filter paper and flash frozen in liquid ethane using a manual plunger. The samples were imaged at liquid nitrogen temperatures on a TF-20 FEG electron microscope (FEI Company) operating at 200 kV and recorded at 25,000x magnification. Images were collected on a Tvips Tietz 4k × 4k CMOS-based camera under low dose conditions with defocus values ranging from −2 to −6 um.

High-quality images of Drp1-lipid tubes were used to box helical segments and collect particles used for image reconstruction methods (more detail in Supplementary Methods). Particles were sorted based on diameter and homogeneous datasets were used for 3D reconstructions of both structures (10,238 segments of the GC/PS filaments and 9,514 segments of the GC/CL segments). The iterative helical real space reconstruction (IHRSR) method^35^ was used to refine the structures. The final GC/PS map converged to a rotation angle of 35.9° per subunit (~10 subunits per turn) and rise of 12.7 Å per subunit (a helical pitch of ~127 Å) with a final resolution of 20.8 Å. The final GC/CL map converged to a rotation angle of 39.9° per subunit (~9 subunits per turn) and rise of 14.4 Å per subunit (helical pitch of ~130 Å), and the resolution of the final map was determined to be 21.0 Å. Pseudoatomic models were built for the GC/PS and GC/CL structures by docking X-ray structures of Drp1^18, 20^ to the EM density via methods described previously^16^ (more detail in Supplementary Methods).

### Sedimentation Assay

To quantify Drp1 oligomerization, a sedimentation assay was conducted similar to what has been described previously^9, 53^. Large oligomers formed by Drp1 samples, in the presence of ligands, were found in the pellet after a medium speed centrifugation. Specifically, protein was diluted in PBS buffer to 0.05 mg/ml (0.62 µM), and specified WT and ∆VD mutant samples were incubated at room temperature with lipid nanotubes (200 µM) and/or GMPPCP (1 mM) for at least 30 minutes. The mixtures were then spun at 13,200 rpm (16,100 × *g*) for 30 min in a tabletop centrifuge (Eppendorf). The supernatant and pellet fractions were separated, collected, and immediately mixed with SDS-PAGE loading dye (Bio-Rad) and heated briefly at 100 °C. These samples were run on an SDS-PAGE gel and stained with an InstantBlue Coomassie dye (Expedeon). Gels were scanned (HP Scanjet 8300) and densitometry analysis was done using the ImageJ software^54^.

### GTPase Assay

Drp1 GTPase activity was determined using a colorimetric phosphate generation assay with some modifications^38^. Briefly, Drp1 (0.5 μM final) was diluted to 1.2x with Assembly Buffer (25 mM Hepes KOH, 150 mM KCl, pH 7.5) in the presence or absence of lipid nanotubes (200 μM total lipid final) for 15 minutes at room temperature. 3x GTP/Mg^2+^ (1 mM and 2 mM final, respectively) in Assembly Buffer was added to Drp1/lipid mixtures to start reactions and samples were incubated at 37 °C. At designated timepoints, EDTA (0.1 M final) was added to sample aliquots. Malachite green reagent (1 mM malachite green carbinol, 10 mM ammonium molybdate tetrahydrate, and 1 N HCl) was added to each sample and A_650_ was measured using a Versamax microplate reader (Molecular Devices). Using Excel (Microsoft), the obtained raw phosphate levels were converted into rates using all data that contributed to a linear trend. These rates were converted to k_cat_ by accounting for Drp1 concentration and were plotted in GraphPad Prism 6. Statistical significance was determined using an unpaired t-test.

## Electronic supplementary material

Supplementary Information
