# Supplementary material for: Cryo-EM Studies of Drp1 Reveal Cardiolipin Interactions that Activate the Helical Oligomer
Source: Sci Rep. 2017 Sep 6;7:10744. doi: 10.1038/s41598-017-11008-3 (PMC5587723; doi:10.1038/s41598-017-11008-3)
Supplement: Supplementary file 1 — Supplementary Information [file 41598_2017_11008_MOESM1_ESM.pdf]

Supplementary Information

Cryo-EM Studies of Drp1 Reveal Cardiolipin Interactions that Activate the Helical Oligomer

Christopher A. Francy [1, 2, 3], Ryan W. Clinton [1, 2, 3], Chris Fröhlich [4, 5], Colleen Murphy[1] and Jason A. Mears[1, 2, 3 ¶]

From the [1]Department of Pharmacology, [2]Center for Mitochondrial Diseases, the [3]Cleveland Center for Membrane and Structural Biology, Case Western Reserve University School of Medicine, Cleveland, OH, 44106, [4]Max-Delbruck-Center for Molecular Medicine, Berlin, Germany, Chemistry and Biochemistry, [5]Free University Berlin, Berlin, Germany

[¶] Correspondence should be addressed to J.A.M. (e-mail: jason.mears@case.edu)

SUPPLEMENTARY METHODS

Expression and Purification of Drp1

Human Drp1 (UniProtID: O00429-3, residues 1-710, isoform 2) was expressed and purified as described previously[20]. Briefly, Drp1 was cloned into a modified pET28a vector as an N-terminal His_6-tag fusion followed by a PreScission cleavage site, and specified mutations were inserted using standard protocols. The VD (aa 514–613) was deleted as previously described[55]. All constructs were expressed in *Escherichia coli* host strain BL21 DE3 Rosetta2 (Novagen). Bacteria were cultured in TB medium at 37 °C to an OD_{600} of about 0.4 followed by a temperature shift to 18 °C. The protein was expressed for 18 h by addition of 40 μM isopropyl β-D-1-thiogalactopyranoside (IPTG). Bacteria were collected by centrifugation and resuspended in buffer A (50 mM HEPES/NaOH (pH 7.5), 400 mM NaCl, 5 mM $MgCl_2$, 40 mM imidazole, 2.5 mM 2-mercaptoethanol, 1 mM DNase (Roche), 100 μM Pefabloc (Roth), followed by cell disruption in a microfluidizer (Microfluidics). Lysates were cleared by centrifugation at 40,000 x *g* for 30 min at 4°C, and the filtered supernatant was applied to a Ni-NTA column pre-equilibrated with buffer B (50 mM HEPES/NaOH (pH 7.5), 400 mM NaCl, 5 mM $MgCl_2$, 40 mM imidazole, 2.5 mM 2-mercaptoethanol). The column was extensively washed with buffer B, followed by buffer C (50 mM HEPES/NaOH (pH 7.5), 800 mM NaCl, 5 mM $MgCl_2$, 40 mM imidazole, 2.5 mM 2-mercaptoethanol, 1 mM ATP, 10 mM KCl) and buffer D (50 mM HEPES/NaOH (pH 7.5), 400 mM NaCl, 5 mM $MgCl_2$, 80 mM imidazole, 2.5 mM 2-mercaptoethanol, 0.5% (w/v) CHAPS). Bound Drp1 was eluted with buffer E (50 mM HEPES/NaOH (pH 7.5), 400 mM NaCl, 5 mM $MgCl_2$,

300 mM imidazole, 2.5 mM 2-mercaptoethanol) and dialysed overnight at 4°C (18 kDa cutoff) against buffer B without imidazole in the presence of PreScission protease to cleave the N-terminal His_6-tag. The protein was re-applied to a Ni-NTA column pre-equilibrated with buffer B without imidazole to which it bound under these conditions also in the absence of the His_6-tag. Subsequently, the protein was eluted with buffer B. In a final step, Drp1 was purified by size-exclusion chromatography on a Superdex-200 column (GE) in buffer F containing 20 mM HEPES/NaOH (pH 7.5), 300 mM NaCl, 2.5 mM $MgCl_2$ and 2.5 mM dithiothreitol. Fractions containing Drp1 were pooled, concentrated and flash frozen in liquid nitrogen.

Hand Determination

To determine the handedness of the Drp1 tubes, negative stain EM samples were imaged at tilts of 0 and -40˚ or -50˚ on a TF-20 FEG electron microscope (FEI Co.) operating at 200 kV and recorded at 25,000x magnification using a TVIPS Tietz 4k x 4k CMOS-based camera. More pronounced striations for the Drp1 filament on GCPS and GCCL were observed on the top of the tube (**Supplementary Fig. 2**), confirming that they are right-handed helices, which is consistent with previous hand determinations for dynamin 1[39] and TMV[56].

3D Reconstruction of Drp1 Lipid Tubes

Images were contrast transfer function corrected using the TOMO-CTF image processing package[57]. Selected Drp1-lipid tubes with good diffraction and minimum astigmatism and drift were initially boxed into helical segments at a width of 180 pixels using the Helixboxer[58] program in the EMAN suite[59]. Projection matching was

implemented using SPIDER software[60] to sort boxed segments based on tube diameter (26,272 total particles for the GC/PS dataset and 26,138 for the GC/CL dataset). For this supervised classification, the particles were aligned to 17 cylindrical diameter classes ranging from 33 to 61 nm. Classes with average diameters centered on ~50 nm (**Supplementary Fig. 2a**) were used for the final 3D reconstructions of both structures. Based on this classification, 10,238 segments of the GC/PS filaments and 9,514 segments of the GC/CL segments were used in the final refinement.

The iterative helical real space reconstruction (IHRSR) method[35] was used to refine the structures. The particles were initially aligned to a featureless cylinder model, and initially, helical parameters for the GC/PS map started with symmetries that were consistent with the geometries identified for dynamin given the similar diameters (i.e. ~50 nm containing ~11-14 subunits per turn in dynamin[16,24,25,39]). This prior knowledge provides an initial estimate of the helical parameters given the similarities in Drp1 and dynamin sequence and structures determined in previous X-ray crystallography studies[20,44,45]. Additionally, we know that the building block of the Drp1 is a dimer[12], which is consistent with previous dynamin structures. The GC/PS dataset correlated better with helical parameters containing fewer subunits per turn, and the reconstructions that started with more subunits were less favored qualitatively as well (i.e. density appeared smeared and artifacts/discontinuity were apparent, which is consistent with poor symmetry alignment). This was a first indication that the spacing was greater for Drp1 polymers when compared with dynamin. For this reason, we tested additional symmetries and settled on 10 subunits per turn. Quantitatively, the data best

aligns to symmetries at or near 10 subunits per turn based on cross-correlation values when aligning the raw data to helical projections. Qualitative assessment further supported this decision, as the map with ~10 subunits per turn was more uniform and the density clearly highlights the GTPase and stalk features of dynamin-related proteins. During refinements, in-plane rotation was limited to ±5˚ and a cross-correlation cutoff was used to remove particles that did not align. For the final reconstruction, the datasets were aligned to 1,890 reference images to account for 4° helical rotations and out-of-plane tilts from +20˚ to -20˚ at 2º increments. The final GC/PS map converged to a rotation angle of 35.9˚ per subunit (~10 subunits per turn) and rise of 12.7 Å per subunit (a helical pitch of ~127 Å, which is consistent with the spacing of the layer lines). The resolution of the final map was determined to be 20.8 Å by Fourier shell correlation and was calculated using the gold standard method[61]. Following from the reconstruction on the PS template, a similar symmetry was initially used for the CL map, and the data converged to a rotation angle of 39.9˚ per subunit (~9 subunits per turn) and rise of 14.4 Å per subunit (helical pitch of ~130 Å), and the resolution of the final map was determined to be 21.0 Å.

Docking All-Atom Structures into Cryo-EM Density

Cryo-EM density was incorporated as a structural restraint to refine the relative positions of Drp1 GTPase and stalk regions using the YUP software package[62]. All-atom Drp1 structures were placed in several distinct orientations within both the GC/PS and GC/CL maps to examine as many conformations that reasonably fit to the EM densities. The Yup.vlat method was used to implement a vector lattice (VLAT) force field

term defines the cryo-EM density as a three dimensional potential, providing a score for fitting the Drp1 structures to the density. Refinements were performed using rigid body Monte Carlo with simulated annealing, which allows for exhaustive sampling of conformational space while the structures move to orientations that best match the cryo-EM data. During these fittings, several reduced-representation models (Cα atoms) of the Drp1 crystal structure[20] were initially evaluated. For the GC/CL map, the GTPase and BSE regions were treated as a rigid unit with the stalk as a separate rigid body to achieve the best fit. From several starting models, the structures converged to a most common fit. This fit was then used to build multiple turns of the helix and a second fitting protocol was run to validate the placement. For the GC/PS map, a similar protocol was used. It became clear that the front-to-front orientation of the GTPase domains was not feasible based both on the lack of fit quality and topological limitations (**Supplementary Fig. 3**). Therefore, the 80 loop structure of the Drp1 GTPase and BSE domains[18] was fitted to the peripheral radial density (green, **Fig. 1d**) and the stalk region from the Drp1 crystal structure used previously[20] was treated as a separate rigid unit. The 80 loop dimer fit the GTPase density of the PS map, and the stalk regions fit into adjacent density diving toward the membrane. To match this topology, the BSE was also reoriented into an “open” conformation, consistent with the GTP-bound conformation seen with Drp1[17] and other dynamin family members previously[16]. Again, a common fit was determined from several starting orientations for both the GTPase-BSE and stalk regions. This fit was then used to build a multiple turns of the helix and a second fitting protocol was run to validate the placement. After the final refinements, all-atom

structures were superposed onto the refined Cα backbones for the Drp1 structures in both the PS and CL maps. The depictions of cryo-EM helical structures and fitted structures were generated using UCSF Chimera[63].

55. Hansson, M. D., Rzeznicka, K., Rosenbäck, M., Hansson, M. & Sirijovski, N. PCR-mediated deletion of plasmid DNA. *Anal Biochem* **375**, 373-375 (2008).
56. Finch, J. T. The hand of the helix of tobacco virus. *J Mol Biol* **66**, 291-294 (1972).
57. Fernández, J. J., Li, S. & Crowther, R. A. CTF determination and correction in electron cryotomography. *Ultramicroscopy* **106**, 587-596 (2006).
58. Ludtke, S. J., Baldwin, P. R. & Chiu, W. EMAN: semiautomated software for high-resolution single-particle reconstructions. *J Struct Biol* **128**, 82-97 (1999).
59. Tang, G. et al. EMAN2: an extensible image processing suite for electron microscopy. *J Struct Biol* **157**, 38-46 (2007).
60. Frank, J. et al. SPIDER and WEB: processing and visualization of images in 3D electron microscopy and related fields. *J Struct Biol* **116**, 190-199 (1996).
61. Henderson, R. Avoiding the pitfalls of single particle cryo-electron microscopy: Einstein from noise. *Proc Natl Acad Sci U S A* **110**, 18037-18041 (2013).
62. Tan, R. K., Devkota, B. & Harvey, S. C. YUP.SCX: coaxing atomic models into medium resolution electron density maps. *J Struct Biol* **163**, 163-174 (2008).
63. Pettersen, E. F. et al. UCSF Chimera--a visualization system for exploratory research and analysis. *J Comput Chem* **25**, 1605-1612 (2004).

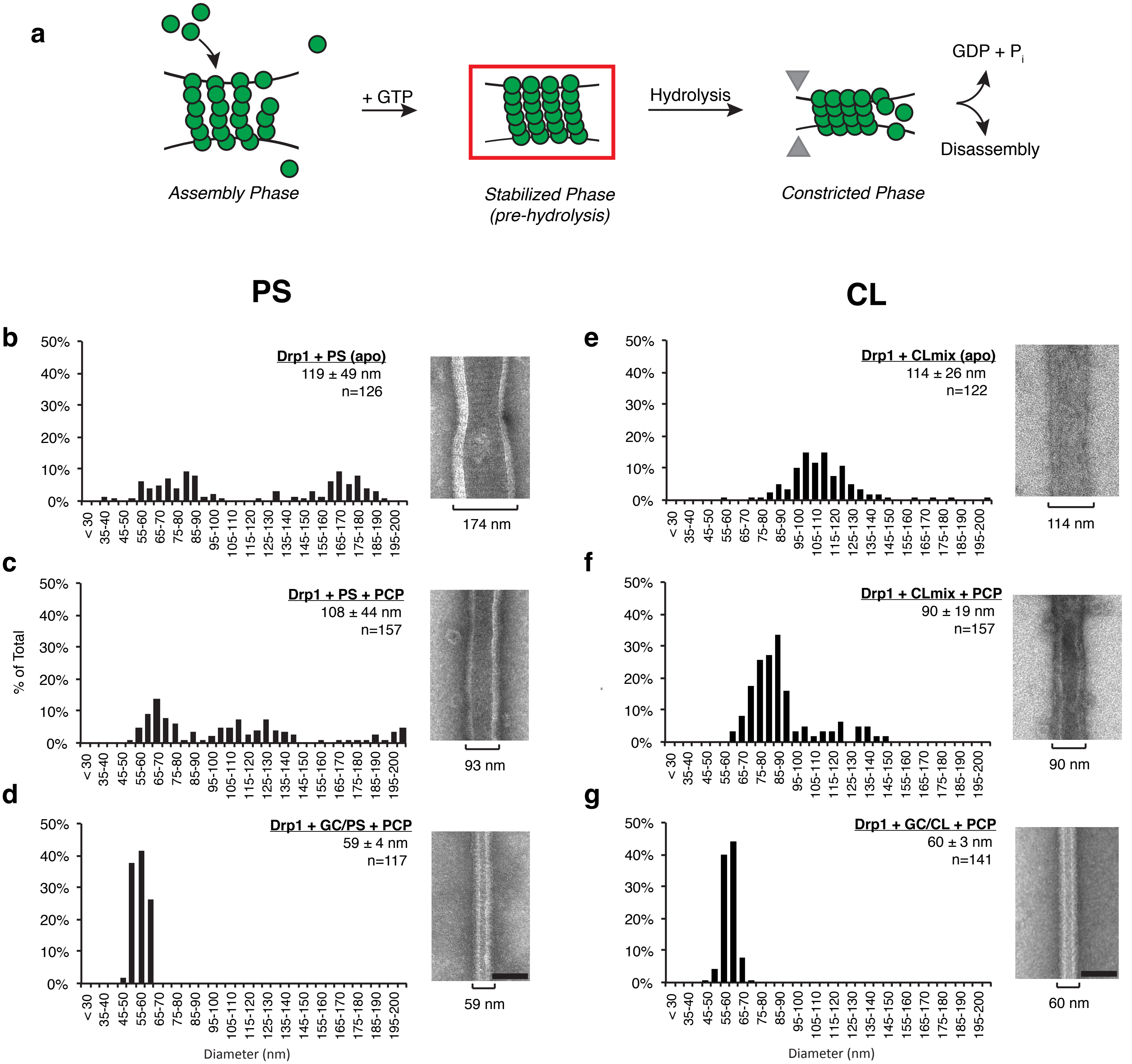

Supplementary Figure 1. Drp1 helical polymers are more uniform on lipid nanotubes in a GTP bound state. (**a**) A schematic demonstrating the constriction cycle of Drp1 (adapted from Francy et al. 2015). Our structures represent the lipid associated GTP bound stabilized phase (red box).The distribution of diameters measured from negative stain EM images are presented for Drp1 helical polymers formed in the presence of PS liposomes (**b**), PS liposomes with GMPPCP (**c**), and PS nanotubes with GMPPCP (**d**). Representative images are shown (right) and diameters are indicated. Comparable data was colleted with CLmix liposomes (**e**), CLmix liposomes with GMPPCP (**f**) and CL nanotubes with GMPPCP (**g**). Scale bar, 100 nm. The average diameter is presented for each sample with the standard deviation and number of measurements (n) indicated.

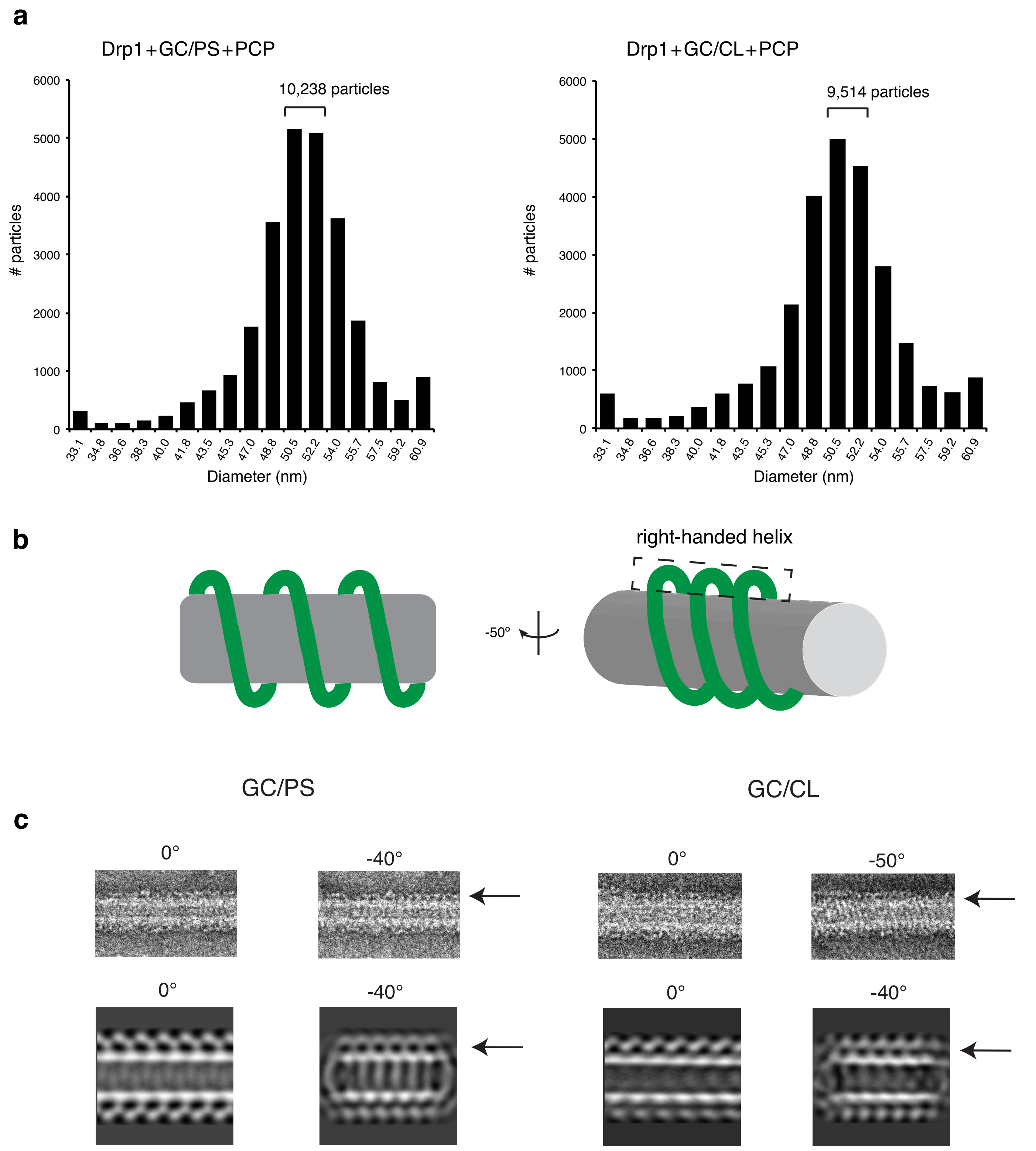

Supplementary Figure 2. Drp1 geometries and handedness used for 3D reconstructions.
(**a**) The diameter distributions of Drp1 helical particles formed on PS and CL nanotubes in the presence of GMPPCP are shown. The two most populated classes were used for helical reconstructions. The total number of particles was 10,236 for the GC/PS sample and 9,514 for the GC/CL sample. (**b**) The handedness of the helical polymers was determined by tilting the sample during image acquisition, and a schematic highlights the changes observed for the helical Drp1 polymer (green) associated with lipid (grey). When a right-handed helix is tilted in the negative direction, the rungs on the top of the helix are more prominent. (**c**) This was observed with 2D projections of the Drp1+GC/PS+GMPPCP and Drp1+GC/CL+GMPPCP helical reconstructions at 0° and -40° tilts. Both experimental samples displayed this phenomenon as well, which indicates that these helical polymers are right-handed.

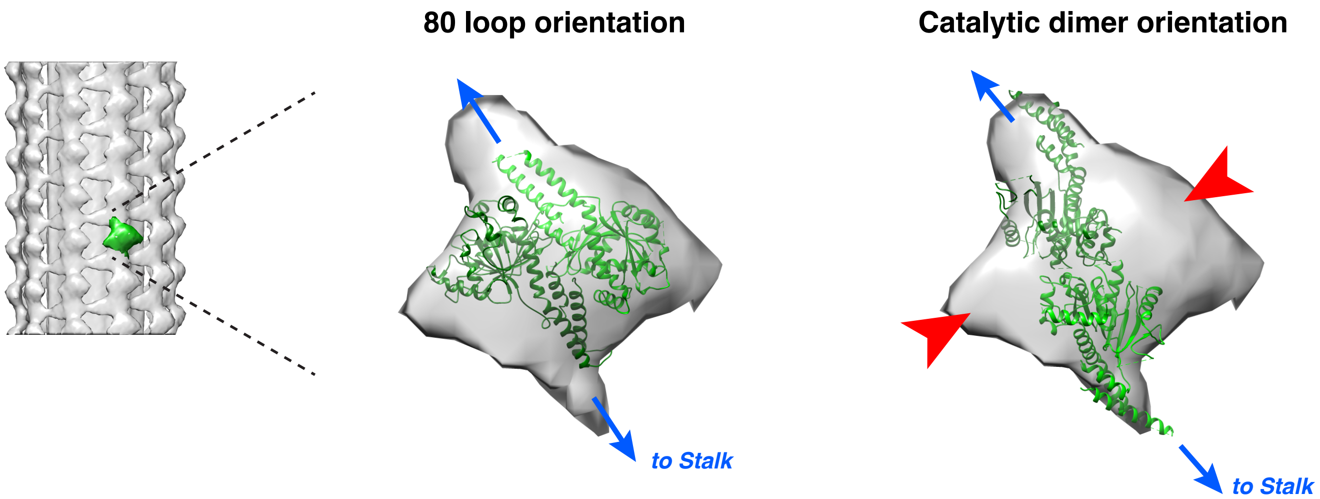

Supplementary Figure 3. Comparison of fitted Drp1 G-domain dimers in the GC/PS density. For orientation, the 3D reconstruction of Drp1 on a GC/PS nanotube is presented with an isolated G-domain density highlighted in green. Distinct G-domain dimers were fitted to this region, and the 80 loop orientation more closely matches the contour of the density. With the catalytic dimer, unoccupied density was observed (red arrowheads).
